# Governance models for cultural assets owned by public healthcare organizations: Italian and Portuguese experiences

**DOI:** 10.3389/fpubh.2026.1953572

**Published:** 2026-09-11

**Authors:** Niccolò Persiani, Giancarlo Landini, Helena José

**Affiliations:** 1 Department of Experimental and Clinical Medicine, University of Florence, Florence, Italy; 2 Fondazione Santa Maria Nuova, Florence, Italy; 3 Nursing Department, Atlântica School of Health, Barcarena, Portugal; 4 Health Sciences Research Unit of Nursing School, University of Coimbra, Coimbra, Portugal

**Keywords:** art therapy, cultural heritage, governance model, healthcare organizations, historical hospitals

## Abstract

**Introduction:**

Healthcare organizations often possess significant cultural assets, accumulated over long periods of activity or through mergers with other healthcare institutions. Several challenges have emerged in conserving and promoting this heritage, including the need for qualified staff, appropriate organizational models, and dedicated financial resources. This study aims to identify a governance model for managing the complex cultural heritage held by healthcare organizations, while always taking into account their primary mission.

**Method:**

To achieve this goal, the paper investigated the experiences of Unidade Local de Saùde de São José in Lisbon (Portugal) and Azienda Unità Sanitaria Locale Toscana Centro in Florence (Italy). These case studies were selected because both entities are characterized by an extraordinary network of historical hospitals that form an integral part of the cultural heritage of their respective cities.

**Results:**

The findings revealed that both case studies followed a similar evolutionary path and ultimately adopted the same governance model already identified in the existing literature.

**Discussion:**

This path could be established by other healthcare organizations with important cultural assets to achieve their dual mission—providing healthcare services while simultaneously preserving and promoting their cultural heritage—because of its total independence to national regulations or healthcare systems' organization. Significant steps by both cases toward the adoption of integrated partnership model suggests that this approach may represent the next stage in the evolution of governance models for cultural assets managed by HOS.

## 1 Introduction

In the pursuit of universal health coverage, healthcare systems worldwide have evolved not only through the construction of modern infrastructures, the acquisition of cutting-edge technologies, and the adoption of economic management strategies (1) but also by tailoring these interventions to local needs, values, behaviors, cultures, and traditions (2, 3).

Although the modernization and innovation are typical features of healthcare facilities, it happens that hospitals, primary healthcare centers, and outpatient services are sometimes housed in historical buildings adorned with artworks and other cultural treasures for maintaining the link with the local context (4).

Over time, these cultural assets have become integral part of the cultural heritage of the cities in which healthcare organizations (HOs) are located due to both their social impact and their artistic and historical significance (5, 6). Moreover, the World Health Organization itself acknowledges the role of art as a powerful tool for promoting wellbeing and healthy lifestyles (7), supporting treatment through art therapy (8–11), and humanizing care environments (12, 13). For this reason, the HOs' cultural heritage can be considered also such as a useful tool for alternative approaches for promoting these interventions.

Health organizations that choose not to abandon or entrust museums with these cultural heritages—often very heterogeneous and composed of historic hospitals, libraries, archives, etc.—must reconcile the challenges related to the provision of health services with that of preserving and enhancing an extraordinary artistic and cultural heritage. This requires specific expertise, resources, and organizational models capable of addressing both key aspects which are currently absent in HOs.

While existing literature has frequently examined the governance structures of such entities and optimal healthcare delivery methods, limited research has tackled the organizational and management models aimed at valorizing the cultural and artistic assets in their possession. This gap highlights the need to identify governance model for managing the complex cultural heritage held by HOs, potentially integrating the strategies for health goals with those for the conservation and promotion of cultural assets.

Accordingly, this study aimed to identify governance models that effectively manage the complex cultural heritage as such historical hospitals held by HOs, facilitating the integration of heritage promotion with the achievement of core healthcare objectives.

## 2 Theoretical framework

According to the Worlds Health Organization's definition of health as “a state of complete physical, mental, and social wellbeing, and not merely the absence of disease or infirmity,” evidence increasingly supports the idea that healthcare requires the implementation of integrated systems. To fulfill this goal, countries should establish effective and equitable integrated social and healthcare systems (14). Healthcare systems have the responsibility of planning, coordinating, and managing the delivery of joint social and healthcare services. It encompasses ensuring equitable access and maintaining high quality across a broad spectrum of services, ranging from preventive and primary care to highly specialized and acute interventions (7, 8, 15). These systems must be capable of accompanying individuals throughout every phase of life, not only during periods of illness, ensuring the promotion of wellness and wellbeing as the continuity of care (9).

The provision of healthcare services is typically entrusted to healthcare organizations (HOs) whose primary mandate is the delivery of care. HOs must be deeply rooted within their communities to identify and address both existing and emerging health needs (10), and optimize performance to deliver high-quality care securing long-term sustainability of healthcare systems (16). In recent years, HOs should implement robust administrative, organizational, and financial strategies to navigate daily challenges in the management of intrinsic complexity of healthcare system (17) such as limited local capacity regarding staffing and expertise (18), and persistent financial pressures (19).

Due to the primary mission of providing care, the value of HOs as cultural heritage has long remained largely unexplored (11, 18). Over time, however HOs have become integral pillars of the cities in which they were founded, owing to their historical and cultural significance within their communities (4). Indeed, many HOs dates back several centuries, having originally been established as religious or civic institutions devoted to hospitality and social welfare, and have continued to serve their communities across generations.

Some of them have accumulated substantial cultural assets comprising artworks, objects of worships, and legacies resulting from their centuries-long activity. Additionally, collections of medical instruments, historical volumes, furnishings, essays, and clinical documents have emerged, bearing witness to storied past. Monumental buildings, churches, and ancient pharmacies have further enriched this heritage. In some cases, older goods and structures—recognized not for their architectural or artistic value but as symbols of pivotal historical events—have survived to the present day (12). Nowadays, these unique and complex cultural heritages, while deeply rooted in the history of healthcare and community services, require thoughtful governance to be preserved and valorized, potentially proving useful in achieving the health mission of HOs (13). Unfortunately, since the resources allocated, expertise available, and organizational models within the HOs fall outside the scope of conservation^1^ and promotion^2^ of cultural heritage (21–24), these critical issues have warranted deeper reflection and analysis on governance model for the management of cultural assets possessed by HOs. The absence of appropriate models has usually resulted in the abandonment, dispersion or the cession of cultural to separate cultural institutions or museums with their exclusion from the HOs' domain (25, 26) thereby severing their connection to healthcare functions. Other time, many historical hospitals have been transformed into museums or cultural centers with the advent of adaptive reuse—as process that repurposes historical buildings for new functions while preserving their cultural and architectural significance (27, 28).

However, the coexistence of healthcare and cultural heritage objectives has sometimes endured (29).

HOs have fully embraced technological progress, prioritizing efficiency, innovation, and high-tech solutions to build smart systems (30, 31), while administrating and valorising the possessed cultural assets. This dual approach allows for the coexistence of cutting-edge medical technological with the artistic and cultural assets accumulated over centuries.

In the field of cultural heritage management, the literature provided a comprehensive and extensive overview of cultural governance that was investigated by different perspectives in the literature (22, 32, 33); however, only a limited number of studies have proposed models specifically tailored to HOs' needs in reaching the dual purpose of delivering care while also valorising cultural heritage (29).

As investigated by Giusti et al. (29), the models identified across different contexts can basically be classified into four types:

(a) HOs managing their cultural heritage directly (internal administration);
(b) HOs enhancing it through dedicated entities (autonomous entities);
(c) HOs operating through networks with other cultural institutions (cultural holdings);
(d) HOs establishing networks with private entities (integrated partnerships).

According to the same authors, these do not represent four separate and alternative models, but are rather interconnected in an evolutionary path that unfolds alongside a growing awareness of the coexisting dual goals and the resulting management complexity.

## 3 Materials and methods

A qualitative multiple-case study was deemed the most appropriate approach for achieving the scope of this study. This approach enables a systematic comparison across multiple cases, allowing both the identification of recurring patterns and the recognition of context-specific variations (34, 35). By examining similarities and differences among cases, it became possible to generate more robust and transferable insights, strengthening the explanatory power of the analysis. Moreover, cross-case analysis facilitated a deeper understanding of complex phenomena as they unfold within their real-world contexts—an essential feature when investigating governance models designed to balance and integrate multiple institutional objectives (36).

Overall, the cross-case analytical framework provided a comprehensive lens through which the interplay between healthcare performance and cultural heritage valorization can be systematically examined (37).

Two significant case studies were selected and analyzed through a cross-case comparison: the Unidade Local de Saùde de São José in Lisbon—ULS SJ (Portugal) and the Azienda Unità Sanitaria Locale Toscana Centro in Florence—AUSL TC (Italy). The choice of these two case studies was guided by a clearly defined comparative logic aimed at strengthening the analytical robustness of the study.

### 3.1 Case studies

Rather than representing isolated examples, these cases were purposefully chosen to enable a structured comparison between contexts that are similar along key dimensions, while also exhibiting meaningful differences relevant to the research objectives. More specifically, both HOs are embedded in historically and culturally significant urban contexts—Lisbon and Florence—which have played a pivotal role in the artistic, cultural, and scientific development of their respective countries. These cities provide a particularly suitable setting for the study, as they host longstanding healthcare institutions that are deeply intertwined with cultural heritage. In both cases, hospitals are not only sites of care delivery but also repositories of historical memory, housing artworks, monumental architectures, and archival materials of considerable value (23, 24).

This shared characteristic ensured a common analytical ground for examining how healthcare and cultural functions coexist and interact. At the same time, each HO is responsible for managing a complex and distributed cultural heritage, including collections of artworks, historical medical instruments, clinical documentation, rare books, monumental buildings, churches, and ancient pharmacies (38–40). These assets are embedded within a network of healthcare facilities characterized by diverse histories and management needs. The coexistence of such multifaceted heritage within operational healthcare organizations made these cases particularly suitable for investigating how governance models can effectively integrate care delivery with cultural preservation and valorization. In fact, the two case studies offered variation in governance models, organizational practices, and approaches to heritage management, thereby allowing the study to capture different responses to similar challenges. This balance between comparability and heterogeneity was central to the cross-case design, as it enabled the identification of both convergent patterns and context-specific dynamics. In the specific case of HOs, this method proved particularly valuable, as it allowed researchers to explore how organizational structures, decision-making processes, and stakeholder interactions contribute to the simultaneous maximization of healthcare delivery and cultural functions (41). In addition, the approach supported the triangulation of findings by combining evidence from different cases, thereby enhancing the validity and reliability of the results. It also enabled the identification of best practices and critical challenges, offering a nuanced perspective on how governance models can be adapted or replicated across different settings (42). Furthermore, both HOs operate as public entities within their respective national healthcare systems and are funded through general taxation. This institutional alignment ensured that their primary mandate remains focused on healthcare provision, with cultural heritage management representing an additional, and often secondary, responsibility. Their comparability was further reinforced by similarities in terms of catchment population, workforce size, and healthcare infrastructure, which helped control for structural differences that might otherwise affected the analysis.

### 3.2 Methods

**Figure 1:**
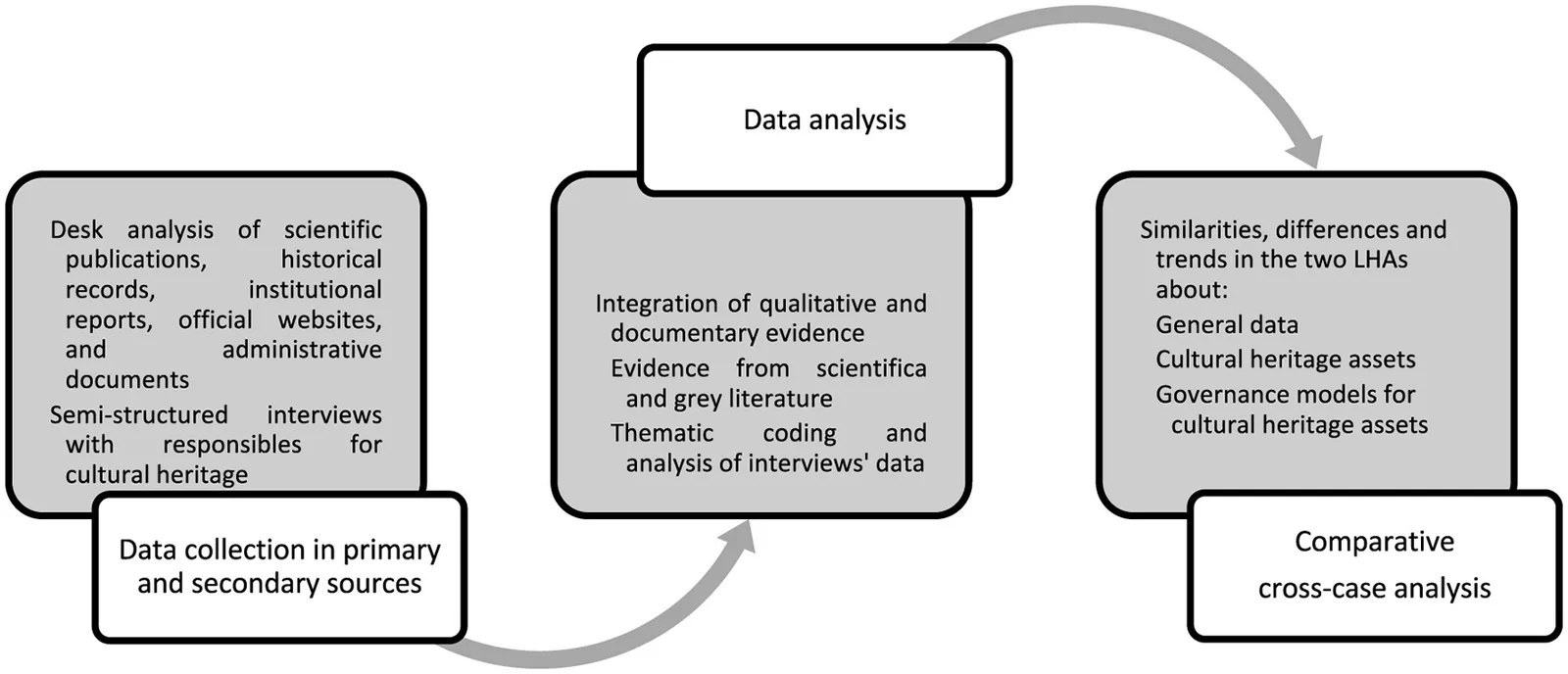
Scheme of applied mixed methods.

The investigation was conducted in three main phases, following a structured qualitative research design consistent with established approaches in case study analysis (43, 44) (Figure 1).

For each case study, data collection was carried out through a combination of primary and secondary sources to ensure methodological triangulation and enhance the robustness of the findings. First, documentary analysis of primary sources was conducted, including scientific publications, historical records, institutional reports, official websites, and administrative documents. This desk-based research enabled the reconstruction of the historical development, institutional structure, and cultural heritage assets of each Local Health Authority (LHA), while also providing a contextual foundation for the subsequent phases of analysis.

After documents' desk-analysis, semi-structured interviews were designed to complement and validate findings emerging on the organizational and governance structures adopted for cultural heritage management and to capture experts' perspectives on challenges, strategies, and future directions. The interview protocol was developed based on the research objectives and informed by prior literature on historical hospitals (29). So, the framework for interview was structured into three main thematic sections:

1. General information about the HO focusing on historical background, institutional evolution, and current organizational structure.
2. Cultural heritage assets, including their identification, conservation, and promotion.
3. Governance model of HO's cultural heritage assets, with particular attention to decision-making processes, stakeholder involvement, and management practices.

Each section included core questions asked to all participants, alongside follow-up probes tailored to the specific context. This structure ensured both consistency across cases and the possibility to explore emerging issues in depth.

Interviews were conducted with responsible for conservation and promotion of cultural heritage of each HO.

These constituted a purposive key-informant sample capable of effectively representing the two different scenarios.

Interviewees were purposively selected based on their institutional roles and expertise. In Lisbon, Dr. C.A. was interviewed as representative of the Cultural Heritage Office of the ULS SJ, while in Florence Dr. C.B., Secretary of the Santa Maria Nuova Foundation established by the AUSL TC, was interviewed.

All participants provided informed consent to take part in the study, to the processing of the collected data, and to their use for research purposes, in accordance with applicable ethical principles and the General Data Protection Regulation (GDPR—Regulation (EU) 2016/679).

Interviews were audio-recorded (with consent), transcribed verbatim, and analyzed using a thematic analysis approach. The analysis followed a coding multi-step process (45, 46):

- Familiarization: transcripts were carefully read to gain an overall understanding of the content.
- Initial coding: relevant excerpts were coded inductively and deductively, based on both emerging themes and the predefined analytical framework.
- Thematic categorization: codes were grouped into broader thematic categories corresponding to the four main dimensions of the interview framework.
- Interpretation: themes were interpreted in relation to the research objectives, with particular attention to governance models and cultural heritage management practices.

Each case study was analyzed independently using the same analytical protocol to ensure a complete and unbiased overview. This structured approach allowed for the systematic extraction of both qualitative insights and descriptive information. The use of a common coding framework ensured consistency across the two case studies while preserving context-specific nuances. Findings from documentary analysis and interviews were integrated to provide a comprehensive understanding of each case. To enhance validity and reduce interpretative bias, a respondent validation process (member checking) was conducted: preliminary findings were shared with interviewees to confirm accuracy and interpretation (47).

Following the within-case analysis, a comparative cross-case analysis was conducted to identify similarities and differences between the selected case studies. This approach enabled the systematic comparison of governance models while preserving the contextual specificity of each case. The analysis focused on identifying recurring patterns, divergences, and critical factors influencing the management of cultural heritage (48). Based on these findings, the study explored the potential for analytical generalization and discussed how the results may inform broader governance strategies for cultural heritage within HOs internationally.

## 4 Case studies

### 4.1 Unidade Local de Saùde de São José in Lisbon—ULS SJ (Portugal)

The history of the Portuguese hospital of São José dates to the reign of King João II. In 1492, the foundation stone of the Real Hospital of *Todos-os-Santos* was laid in Rossio, and the hospital was officially inaugurated by King Manuel I in 1504. Following the devastating earthquake of 1755, the hospital was relocated to the former Convent of Saint Antão and renamed the Real Hospital of *São José*. Over time, it incorporated other hospitals—hospitals *Desterro, Arroios*, and *D. Estefânia*—forming what become known as the *Real Hospital de São José* and Annexes (49). Subsequently, other long-established hospitals in Lisbon, including hospitals of *Santa Marta, Curry Cabral (Rego)*, and *Santo António dos Capuchos*, were integrated (24).

With the proclamation of the Republic in 1913, the established network of hospitals was renamed the *Civil Hospital* of Lisbon, a designation that has evolved into the current *University Hospital Center of Lisbon* (CHULC), established by the Decree-Law No. 61/2018. Today, CHULC comprises six hospitals: Hospital of *São José* (HSJ), Hospital of *Santo António dos Capuchos* (HSAC), Hospital of *Santa Marta* (HSM), Hospital of *Dona Estefânia* (HDE), Hospital of *Curry Cabral* (HCC) and Maternity Hospital of *Dr. Alfredo da Costa* (MAC). These institutions collectively represent a rich and layered cultural heritage, while continuing to serve as active healthcare providers within Portugal's national health system.

Spanning more than five centuries, the former CHULC preserves a rich cultural asset, which includes historical hospitals, churches, fountains, cloisters, and an exquisite collection of *azulejos* that enrich the cultural and historical significance of the site (38). For centuries, this complex has played a central role in clinical education in Portugal, contributing to the development of medical knowledge and professional training (50). It also houses collections of medical instruments, ancient archives, and libraries as legacy of the medical traditions of the CHULC, accessible either freely or by appointment attending visits to hospitals or to the Health Museum hosted there.

The Unidade Local de Saùde de São José in Lisbon—ULS SJ was established by Decree-Law No. 102/2023 on November 7th, 2023, and officially began operations on January 1st, 2024. This new entity resulted from the merger of several institutions: the CHULC, the Hospital *Júlio de Matos* Hospital—Psychiatric Hospital Center of Lisbon (CHPL), the Institute of Ophthalmology *Dr. Gama Pinto* (IOGP), the Health Center Groups of *Lisboa Central* (ACES Lisboa Central), and the Health Center Unit *Sacavém* (CS Sacavém) previously part of the Health Center Group *Loures-Odivelas* (ACES Loures-Odivelas). LHA JS now provides hospital care across nine different locations and operates an integrated primary healthcare structure compromising 33 functional units. These include Family Health Units (USF), Personalized Health Care Units (UCSP), Community Care Units (UCC), and Public Health Units (USP), reflecting a comprehensive and decentralized approach to healthcare delivery (Table 1).

**Table 1:** Comparison of selected case studies.

Areas	USL São José		AUSL Toscana Centro	
*Structures*	Hospitals	11 with more than 1,500 beds	Hospitals	13 with 2,735 beds
	Family Health Units Personalized s Health Care Units Community Care Units and Public Health Unit	33	District Zones	8
			Health Companies	8
			Territorial Facilities	220
*History*	1492—Foundation stone of the Real Hospital de *Todos-os-Santos* in Rossio 1504—Inauguration by King Manuel I in 1755—Relocation to the former Convent of *Santo Antão* and renamed the Real Hospital of *São José* and Annexes, incorporating Hospitals of *Desterro, Arroios*, and *D. Estefânia* 19th century—Incorporation of other long-established hospitals in Lisbon, such as *Santa Marta*-−1569, *Curry Cabral (Rego)*, and *Santo António dos Capuchos—*1836 1913—Civil Hospital of Lisbon 2018—University Hospital Center of Lisbon, incorporating also the Maternity Hospital of *Dr. Alfredo da Costa* 2024—Establishment of Local Health Unit São José		1288—Foundation of Hospital of *Santa Maria Nuova* 15th−16th century—Hospital of *Santa Maria Nuova* as center of scientific research and incubator of the artistic and cultural knowledge 17th−18th centuries—Santa Maria Nuova Hospital as example at national and international level for the canons of health care 18th−19th centuries—Organizational and managerial difficulties brought to the building of new pavilions outside the city center with the foundation of *Santa Maria Nuova Arcispedale* (University Children *Meyer* in 1884; then the University Hospital *Careggi* in 1910–1930). 1980—Local Health Authority 8 of Florence 2016—Establishment of the Local Health Authority Toscana Centro	
*Cultural heritage*	- Hospital of *São José* in Lisbon (1492) - Hospital of *Santa Marta* with the Old Church of the Convent of *Santa Marta* in Lisbon (1569) - Hospital of *Santo António dos Capuchos* in Lisbon (1836) - Collection of Azulejos		- Hospital of *Santa Maria Nuova* in Florence (1288) - Hospital of *Ceppo* in Pistoia (1277) - Hospital *Misericordia e Dolce* in Prato (1545) - Hospital *Serristori* in Figline (14th century) - Renaissance art crafts	

The goods that compose the cultural asset of the USL SJ are all inventoried and mainly hosted in the Hospitals of *São José, Santo António dos Capuchos*, and *Santa Marta*, which form a network of historical hospitals. These hospitals have been officially recognized by Portuguese national legislation:

- The main building of the Hospital of *São José*, formerly the College of *Saint Antão-o-Novo*, was classified as a property of public interest by the Decree No. 8/83 of January 24th, 1983, while the former Sacristy of the Church of *Santo Antão-o-Novo* was classified as a national monument by the Decree No. 22/502 of May 10th, 1933, both enacted by the Portuguese Ministry of Public Education, General Directorate of Higher Education and Fine Arts.
- The Hospital of *Santa Marta*, with the Old Church of the Convent of *Santa Marta* was classified as property of public interest by the Decree No. 35/532 on March 15th, 1946, enacted by the Portuguese Ministry of National Education, General Directorate of Higher Education and Fine Arts.
- The Hospital of *Santo António dos Capuchos*, was classified as properties of public interest by the Decree No. 1/93 on January 15rd, 1993, enacted by the Ministry of Culture, Portuguese Institute of Cultural Heritage. This classification includes the former Church of the Convent of *Capuchos*, the mouth of the tile-covered cistern in one of the Hospital's courtyards, and all the rooms decorated with tile paneling, including the cloister and the grand staircase.

Within ULS SJ, a notable cultural asset is the collection housed in the Portuguese Dermatology Museum of *Dr. Lu*í*s de Sá Penella*, located in the Hospital of *Santo António dos Capuchos*. This collection includes a series of wax figures and a documentary archive that spans from the 19th century to the mid-20th century. These items represent a unique intersection of medical history and artistic craftmanship. The idea of establishing dermatology museums in Portugal dates to the 1940s. In 1955, a room at the Hospital of *Desterro* was designated as the Portuguese Dermatology Museum. The museum had two primary objectives: to honor the legacy of dermatologist Dr. Luís de Sá Penella, and to preserve a collection of wax figures depicting various dermatological pathologies. Following the closure of the Hospital of *Desterro* in 2007, the collections was transferred to the Hospital of *Santo António dos Capuchos*. In 2009, the entire collection was brought together in the Noble Room of the hospital, ensuring its continued preservation and public accessibility.

The cataloging of *azulejos* tile decorations across the healthcare facilities of USL JS is currently underway. These artworks are distributed throughout a wide variety of architectural contexts, including cloisters, canteens, wards, churches, stairways, and other spaces. The collection is of exceptional cultural and artistic significance, spanning a remarkable chronological range—from early examples dating back to the 13th century, when *azulejo*s were first introduced in Portugal, to contemporary pieces. Considered as a whole, this collection constitutes a unique and unparalleled corpus, distinguished by its historical continuity and its artistic diversity (51) (Table 1).

### 4.2 Azienda Unità Sanitaria Locale Toscana Centro in Florence—AUSL TC (Italy)

The Hospital of *Santa Maria Nuova* (HSMN) in Florence (Italy) is considered one of the oldest hospitals in the world still operating at its original location (39). Founded in 1288 by a wealthy merchant Folco Portinari—father of Beatriche, the muse of Dante Alighieri—the hospital was established as a philanthropic gift to the city of Florence, aimed at supporting its social and urban development. This hospital quickly became a pioneering model of civic healthcare, inspiring the creation of major hospitals across Europe between the XV and the XVII centuries (9, 23). The HSMN became an example at national and international level for the canons of care used in personal care, the effectiveness of the care provided, and for the efficient organization and management of the structure. Over the centuries, this hospital expanded thanks to generous contributions from nobles, monarchs, and Popes, who recognized its vital role in promoting public health and urban growth.

Between the IXX and the XX centuries, however, the first organizational and managerial difficulties of the structure began to emerge, no longer able to respond to the new building standards set for health care facilities and the health needs' increasing of a city in strong expansion. Due the lack of space within the city center, the SMNH relocated the construction of new pavilions to the Florence's suburbs with the establishment of the *Arcispedale di Santa Maria Nuova e Stabilimenti riuniti*. These hospitals have become the current university hospitals of Florence: University Children Hospital *Meyer* (1884) and University Hospital *Careggi* (1910–1930).

In 1980, the HSMN and the hospitals under its jurisdiction lost their autonomous legal personality, being acquired by the Local Health Units No. 8 of the Florentine area, which was transformed in the Local Health Authority by Legislative Decree 502/92. Today, HSMN is one of the 13 hospitals managed by the Azienda Unità Sanitaria Locale Toscana Centro in Florence—AUSL TC established through the merge of local HOs of Florence, Prato, Pistoia, and Empoli by the Regional Law No. 84/2015. Located in the heart of Florence's historic center-designated a UNESCO World Heritage Site since 1982, the HSMN is a modern hospital housed within a medieval building (52) (Table 1).

The historical and artistic heritage of AUSL TC comprises both immovable assets—monumental complexes of notable historical and architectural importance—and movable assets of indisputable artistic and cultural value, encompassing a wide range of objects: from art collections (paintings, sculptures, tiles, bas-reliefs, etc.) to sacred furnishings, from rare book collections to surgical instruments. Below special attention is paid to the most emblematic goods:

- The Hospital of *Santa Maria Nuova*—*HSMN* (FI). Beyond its historical importance, it boasts a remarkable artistic heritage thanks to the contributions of some of the greatest Florentine artists. Notable features include the facade with loggia by Buontalenti, the cycle of frescoes dedicated to the “Storie dalla vita di Cristo” by Pomarancio, as masterpieces such as “Crocifissione e Santi” by Andrea del Castagno, the tabernacle of the Church of *Sant'Egidio* created by the joint work of Bernardo Rossellino and Lorenzo Ghiberti, the glazed terracotta “Madonna con Bambino” by Andrea della Robbia, and the crucifix by Francesco da San Gallo (23, 53).
- The Hospital *Misericordia e Dolce*—*HMD* in Prato (PO) dates to 1545, when Cosimo I de' Medici ordered the merge of the city's Hospital *Misericordia* and the Hospital of *San Silvestro* of XIII century. Over the centuries, the hospital's governance shifted between different civil institutions, when Pietro Leopoldo retuned control to the Community of Prato in 1788. In 1895, the Hospital *Misericordia e Dolce* ceased its role as a foundling hospital, which childcare responsibilities transferred to specialized institutions facilities (54).
- The Hospital of *Ceppo-HC* in Pistoia (PT), traditionally founded in 1277, served healthcare functions for over seven centuries until the opening of the new city hospital in 2013. Today, it houses a museum itinerary that narrates its historical, architectural, and artistic evolution and some outpatients' services of the AUSL TC that are still active within the complex. A highlight of the collection is the polychrome glazed terracotta frieze by Giovanni della Robbia (1525), which adorns the external loggia and depicts the “Sette opere di Carità”—a masterpiece of Renaissance sculpture (55).
- The Hospital *Serristori*—*HS* in Figline Valdarno (FI) is notable for its elegant interiors and historic pharmacy. It was founded in the late 14th century through a bequest from Count Umberto Serristori. Of the three buildings that comprise the hospital, the most important is the original Villa San Cerbone, later expanded to incorporate the convent of the Sisters *Serve di Maria* congregation. Inside the hospital lies the Ancient Apothecary, where period furnishings, ceramics, and glassware are carefully preserved (56).

All heritage assets have been cataloged and secured to prevent loss or damage due to neglect. Currently, a new comprehensive cataloging initiative is underway, in collaboration with some working groups of the University of Florence. This project, which began at HSMN, is gradually being extended to all facilities of AUSL TC: 13 hospital facilities, 220 territorial structures, eight District Zones, and seven Health Societies, as well as properties no longer directly used for the healthcare activities, all distributed across the provinces of Florence, Prato, Pistoia, and Empoli (Table 1).

## 5 Results and discussion

### 5.1 Governance model for cultural heritage assets in USL SJ

In Portugal the cultural heritage management is regulated by the Decree-Law No. 78/2023. This Decree reorganized the Directorate-General for Cultural Heritage (DGPC) establishing the Cultural Heritage Institute in each public entity, a new public institute responsible for managing national cultural assets.

The DGPC is responsible with fulfilling the State's obligation in safeguarding, research, conservation, management, restoration, enhancement, dissemination, and internationalization of national both immovable and intangible cultural heritage. Operating under the Ministry of Culture, the DGPC allows for a strategic repositioning of asset management, emphasizing greater operational efficiency, the application of regulatory standards and the modernization of technical resources retaining talent and improving the overall functionality of heritage service. This Decree emphasizes the inalienable and cross-cutting responsibility of the State in safeguarding cultural heritage.

To fulfill its mission, the DGPC adherences to the principles outlines in Law No. 107/2001, which defines the policy and the legal regime for the protection and enhancement of cultural heritage, along with other applicable legislation. This legal framework ensures that cultural heritage management is aligned with national priorities and international best practices.

This new regulatory framework presupposes the existence of a decentralized public Institute with legal personality and with administrative, financial, and patrimonial autonomy integrated into the indirect administration of the State. This Institute operates under the supervision and guardianship of the Government member responsible for culture, ensuring both agility in action and effective management of the national cultural heritage.

In accordance with the current regulatory framework, the ULS SJ has established a specific unit named Cultural Heritage Office. This Office replaced the former Cultural Heritage Commission of the CHULC and is tasked with the conservation and promotion of the cultural heritage owned by ULS SJ. Currently, the Office is staffed by a single employer, Dr. C.A.-An historian, specialized in archaeology-, who has served as Director since 2022, after the retirement of the former President of the CHULC's Cultural Heritage Commission.

The main activities of Cultural Heritage Office include:

- tours within the historical hospitals of ULS SJ and exhibitions staged directly into the wards, making the cultural heritage accessible to both patients and visitors. These visits are offered on weekdays, weekends, and holidays, with an average duration of 3 h.
- educational and promotional initiatives. The Office organizes seminars focused on the cultural heritage of LHA SJ and coordinates outreach activities to engage a broader audience. These include guided tours during the University of Lisbon Faculty of Medicine's open days and the provision of hospital spaces for filming television series.

Nowadays, the Office is mainly focused on the promotion of cultural heritage through a strategic approach that prioritizes raising public awareness. The objective is to enhance knowledge of the USL JS's heritage among the public, including Lisbon residents and, increasingly, tourists who have shown growing interest in the city in recent years. Over time, this strategy aims to position tours of USL JS's historical hospitals as a meaningful and alternative way to explore Lisbon's history and identity. By offering visitors the opportunity to discover the city through its hospitals, the Office presents an unconventional tourist route that winds through the heart of Lisbon. This initiative not only highlights the historical and cultural significance of these healthcare institutions but also reinforces their role as living monuments within the urban landscape.

At the same time, recognizing inventorying as a top priority for safeguarding USL SJ's cultural heritage—and acknowledging the shortage of qualified professionals to undertake such work—partnerships have been established in recent years with academic institutions, particularly the University of Lisbon, through formal cooperation agreements. These collaborations remain active and have played a key role in supporting the documentation and study of USL SJ's cultural assets.

Among the strategic projects the Office is currently prioritizing is the recovery and enhancement of the USL JS's *azulejos* collection through the creation of a dedicated interpretive pathway. The historic buildings of LHA SJ contain examples of all major stylistic influences that have shaped the evolution of *azulejos* in Portugal—from their early adoption to their development throughout the XXI century. This diversity enables the design of a comprehensive cultural route that illustrates the history, affirmation, and artistic progression of this iconic decorative technique.

Another priority area, originally initiated by the CHULC Cultural Heritage Commission and subsequently continued by the Office, has been the active engagement of both healthcare professionals and civil society in the conservation, dissemination, and promotion of ULS SJ's cultural heritage. To this end, a range of cultural and outreach initiatives have been organized, including Heritage Colloquia, the European Heritage Days, the International Day for Monuments and Sites, and the Antonian Journeys. These activities have contributed to strengthening public awareness of the institution's heritage assets and fostering a broader culture of participation in their safeguarding and valorization. Nevertheless, the use of the cultural assets for the promotion of initiatives such as the humanization of care or art therapy have never been envisaged.

There is also the interest to get on touch and collaborate with the USL de Santo António in Porto, which have adopted a different governance model for the management of its cultural heritage. Here was established a museum which brings together the history and mission of its constituent institutions, alongside unique buildings dedicated to clinical care and the development of academic and research activities in a museum. The idea is to integrate the two experiences for develop a joint model which could be a standard model at national level, recognized both by the ministry of Culture and the Ministry of Health.

### 5.2 Governance model for cultural heritage assets in AUSL TC

In Italy, the Santa Maria Nuova Foundation ONLUS (now SMN Foundation) was established in April 2015 at the initiative of the top management of the ASL no. 10 of Florence. As no-profit and autonomous entity, the SMN Foundation was created with a double function:

1. To protect, promote, and enhance the inestimable historical, artistic, and cultural heritage exclusively of HSMN, collected over more than eight centuries of activity.
2. To support the health-related objectives of the ASL no. 10 of Florence, including the provision of prevention services, medical assistance, and personal care.

The cultural asset's ownership remains of the ASL. While the management of the cultural heritage has been entrusted to the SMN Foundation.

According to its mission and as outlined in its founding Statute, the SMN Foundation collaborates with public and private entities to promote fundraising activities aimed at supporting its cultural initiatives, financing restoration and maintenance interventions for the cultural heritage under its competence and contributing to clinical research and technical innovation within the ASL.

The SMN Foundation is governed by a Board of Directors—four members, one with the President role, which serves a three-year term-, an Auditor and an Executive Secretary. The Board is responsible for all the SMN Foundation's initiatives—both ordinary and extraordinary—aimed at achieving the objectives outlined in its Statute. It defines and guides the strategic directions of the SMN Foundation's activities, ensuring alignment with its institutional goals. The President, appointed by the General Director of the ASL no. 10 of Florence, serves as the legal representative of the Foundation. The Executive Secretary is responsible for informational, administrative, and organizational matters, overseeing all procedures necessary for the Foundation's day-to-day operations. The Foundation also relies on external collaborators, including a tax consultant and a labor consultant, who provide specialized support for its activities.

In 2018 the SMN Foundation launched a museum itinerary within the HSMN—a virtuous example of integration and mutual enhancement between healthcare services and the protection and promotion of artistic heritage. While in 2020, the SMN Foundation was entrusted with the functions and cultural activities previously managed by the Documentation Center for the History of Healthcare and Assistance with iso-resources.

With the entry into force of the Regional Law 84/2015 on January 1, 2016, the ASL of Firenze, Prato, Pistoia, and Empoli were merged into a single entity, the new AUSL TC.

Following the amendment of its Statute in June 2021, the SMN Foundation broadened its mandate, having been entrusted with the management of the cultural heritage of the AUSL TC. This responsibility arose from the integration, within the new organization, of the cultural assets previously held by each ASL.

Two key changes were introduced in the new Statute of the Foundation:

1. The direct assignment of the AUSL TC's cultural heritage to the SMN Foundation, enabling it to offer donors the tax benefit associated with the “Art Bonus.”^3^ This measure was designed to enhance the SMN Foundation's appeal and encourage greater donations by fostering interest in the heritage and the initiatives aimed at its preservation and restoration.
2. The reinforcement of the Foundation's role in supporting healthcare activities of the AUSL TC: “A mission that grows and renews over time: supporting and ensuring the efficiency of our structure and always standing alongside patients and families in times of need. Our objectives are to continuously improve all processes for the benefit of our stakeholders, with the precise aim of better connecting the Foundation's reality with the categories targeted by our actions—public, private, academic, business, and voluntary sectors—through an ever-stronger bond with the territory.”

After these changes, the SMN Foundation was also qualified as a third sector body (called ETS).

About healthcare purposes, following the COVID-19 pandemic, the SMN Foundation partially redirected its activities to support purely medical purposes by launching fundraising campaigns in favor of the AUSL TC.

The key factors of the SMN Foundation lie in its dual nature. On one hand, it operates as an independent entity, free from the constraints of public administration allowing it to respond more effectively to emerging needs, and to pursue its strategic objectives with greater agility. On the other hand, it maintains a cultural and institutional connection with AUSL TC, embracing not only the mission of protecting and enhancing cultural assets but also actively promoting health. This dual commitment creates a virtuous cycle, where both cultural and healthcare goals converge toward a shared purpose. In this context, the SMN Foundation has assumed a leading role in fundraising, becoming a point of reference for both the community and the same AUSL TC.

Since 2020, the SMN Foundation is part of the national Cultural Association of Italian Historical Hospitals (ACOSI), of which is founding members. Into ACOSI, SMA Foundation contributes to develop management, conservation, and communication solutions for Italian historical hospital, which can be of example as for similar realties in other countries. The ACOSI association represents the most organized Italian initiative in managing the cultural heritage of historic hospitals, bringing together the vast majority of Italy's ancient hospitals. To learn more about the number, volume, and quality of historical Italian hospital complexes, please visit www.acosi.org, where the association's position paper on the concept of the historic hospital—titled “L'ospedale storico del futuro”—is also available.

### 5.3 Comparison of adopted governance model in case studies

The comparison between the two case studies revealed the adoption of a similar path to identify its own proper governance model (40). In both cases, the management of cultural assets initially felt under the responsibility of HO's heritage office (internal administration), whose role was mainly limited to custodial and administrative functions. However, one these assets were recognized as cultural goods to be preserved and valorized—formally in Portugal through Decree-Law No. 78/2023 and informally in Italy trough the autonomous decision of the AUSL TC board—both organizations established dedicated structures specifically tasked with their valorization and promotion (autonomous entity).

A further step in this evolution emerged when both HOs faced the challenge of managing networks of historic hospitals rather than isolated heritage sites. In response, both cases revised its governance approach by integrating the management of the individual hospitals into a single cultural holding structure, thereby creating an internal network despite pursuing different institutional objectives. Interestingly, this tendency toward integration at the organizational level mirrors the broader trend identified in the literature (29). The establishment of a cultural holding enabled the optimization of few allocated resources with scale economies, the sharing of expertise and experiences, and greater competitiveness (57). These elements proved essential both for strengthening the visibility of historic hospital networks within cultural and tourist itineraries and for increasing their attractiveness to philanthropic organizations and private donors (21, 58).

Despite these similarities, important differences emerged in the way the cultural holding model was implemented in the two case studies, leading to distinct operational and strategic implications.

In the Portuguese case, the main critical issue faced by the Cultural Heritage Office within the ULS SJ was the rigidity of bureaucratic framework combined with the lack of financial autonomy. As a fully public entity, the Office encountered difficulties in accessing private funding necessary to ensure the sustainable maintenance and enhancement of cultural heritage (27, 28). This approach risked generating inefficiencies in resource allocation and financial flows, a common issue in large public healthcare systems (19, 59). Consequently, discussions with the Ministry of Culture were initiated in order to secure dedicated funding for specific projects. Nevertheless, the establishment of the Cultural Heritage Office represented an important step forward, as it provided institutional legitimacy to the preservation, promotion, and enhancement of USL JS's cultural heritage. However, this dedicated structure became a valuable formal interface with key stakeholders—particularly public entities such as the Ministry of Health, the Ministry of Culture, universities, and research institutions—thereby strengthening the HOs' capacity to pursue their mission of promoting cultural assets as well as an important tool for resource accountability.

Conversely, the Italian case demonstrated how decentralization can be leveraged as a strategic advantage through a hybrid governance model (16). By entrusting the management of the historical hospital network to the SMN foundation under public control, the AUSL TC succeeded in bridging the gap between public mission and managerial flexibility (40). This independence made it possible to establish a dedicated entity focused on cultural activities that would typically fall outside the institutional mandate of HOs. Such flexibility generated several advantages: first, it facilitated access to private financial resources, as the Foundation proved more attractive and accessible to donors thanks to accounting and administrative mechanisms that encourage donations and private contributions; second, it enabled the recruitment of specialized professional expertise often unavailable within HOs; third, it fostered partnerships between healthcare institutions, local authorities, and civil society, which are considered essential for improving community health and wellbeing (14, 17); and finally, it promoted the integration of art and cultural heritage into clinical pathways, transforming the hospital from a purely functional environment into a space of humanized care and cultural participation (60, 61). At the same time, however, this governance model also introduced additional complexities. Preserving the close and inseparable relationship between cultural heritage, healthcare practices, and local communities requires continuous and transparent dialogue with the AUSL TC board in order to ensure coherent strategic planning. Maintaining this alignment is both essential and more challenging within a decentralized governance framework (Table 2).

**Table 2:** Comparison of cultural heritage assets' governance model in the selected LHAs.

Variables	ULS São José	AUSL TC
*Structures*	Cultural Heritage Office	Santa Maria Nuova Foundations ETS
*Governance models*	Institutional model according with Decree-Law No. 78/2023	Corporate model established for decision of the LHA TC
*Nature*	Public	Private
*Cultural assets' property*	Internal (Public)	Internal (Public)
*Cultural assets' management*	Internal (Public)	External (Private)
*Boad*	ULS São José's board	Board of Directors (four, one with the role of President)
*Personnel*	LHA SJ's employer	Auditor, Executive Secretary
*Volunteers' support*	Yes	Yes
*Regulation*	Decree-Law No. 78/2023: Directorate-General for Cultural Heritage and local sections	Statute
*Main activities*	Touristic tours	Fundraising
	Educational initiatives	Visits
*Stakeholders*	Visitors	Visitors
	Schools	Patients and caregivers
		LHA's workforce
*Healthcare purposes*	Yes	No

Beyond organizational differences, the analysis also highlighted a profound divergence in the conceptual role attributed to cultural heritage within healthcare settings. In the Portuguese case, cultural assets were primarily managed as a “corporate collection” (49). As a result, the emphasis remained largely confined to historical preservation and tourism-related activities, without significant integration into clinical practise or preventive healthcare strategies. This institutional rigidity has so far limited the possibility of using cultural heritage as a tool to promote public wellbeing (61, 62), support art therapy initiatives (60, 63, 64), or contribute to the humanization of care (13, 64, 65). Consequently, the main challenge faced by the management remains financial sustainability, which depends heavily on competitive public funding schemes promoted by the Ministry of Culture rather than on synergies with the healthcare sector itself (10, 59).

In contrast, the Italian case model assigned cultural heritage a broader and more integrated mission. The SMN Foundation operated according to a dual mandate that combines cultural conservation with health promotion objectives (29). Acting as a “preferential channel” for community donations, the SMN foundation has revitalized the long-standing tradition of philanthropy of the local context (23, 25). This approach supported a circular allocation of resources in which donations were not confined to a single purpose but could simultaneously finance the restoration of fifteenth-century frescoes and the acquisition of advanced medical technologies. In this way, cultural heritage became directly connected to the improvement and humanization of the hospital environment, reinforcing the relationship between healthcare, culture, and community engagement (65).

Because of these critical issues, the establishment of a cultural holding appeared to represent an intermediate step in the evolutionary pathway of cultural heritage governance models within HOs.

We want underline that both cases studies are, actually, assessing the feasibility of establishing integrated partnerships with other HOs to address these challenges. USL SJ was engaged in discussions with the USL de Santo António in Porto, while AUSL TC was collaborating the other HOs belonging to ACOSI. The scope was to integrate resources, personnel, and expertise to standardize and optimize the cultural heritage management practises within HOs.

### 5.4 Limits and future developments

A potential limitation of this study lies in the absence of standardized criteria for identifying cultural heritage assets owned by healthcare organizations (HOs), as well as in the limited number of cases analyzed, all of which belong to public healthcare systems. Future research could therefore contribute, on the one hand, to the development of shared criteria for the identification of such assets and, on the other hand, to the expansion of comparative analyses to include case studies beyond public national healthcare systems, where different governance models may emerge due to varying institutional and financial contexts.

Further research should also investigate whether the evolution of the two case studies will lead—as already suggested in the literature and partially observed in practice—to the establishment of integrated partnerships with other national and international HOs.

## 6 Conclusions

The scope of this study was to explore governance models for managing the complex cultural heritage held by HOs. The Portuguese case study, the ULS SJ in Lisbon, and the Italian case study, the AUSL TC in Florence, were selected because of their significance and shared characteristics, which make them particularly suitable for comparative analysis.

The findings revealed that both case studies followed a similar evolutionary trajectory and ultimately adopted the same governance approach for the management of historic hospitals: the cultural holding already identified in the literature. At the same time, both cases were assessing the feasibility to adopt integrated partnership for the management of their cultural heritage. This suggested that this model may represent the next stage in the evolution of governance models for cultural assets managed by HOS in accordance with the current literature evidence.

The potential for generalization is significant, as this model can be applied to other HOs that possesses complex cultural assets—including networks of historic hospitals—and demonstrates both awareness of their social value and a commitment to their preservation and promotion.

More broadly, this evolutionary path of governance model could be adopted by many other HOs that manage important cultural assets in comparable institutional contexts and seek to fulfill a dual mission: delivering healthcare services while simultaneously preserving and promoting cultural heritage. Its applicability is reinforced by the fact that it is not dependent on specific national regulations or on the organizational structure of individual healthcare systems. Consequently, the evidence gathered from these two case studies can be considered representative and potentially generalizable at the international level, particularly within public healthcare systems.

Overall, these cases illustrate how HOs can preserve the complementarity between their traditional healthcare function and their distinctive responsibility to safeguard and enhance cultural heritage assets. This role becomes especially relevant when society recognizes hospitals and healthcare facilities not merely as places of care, but also as expressions of collective values, beliefs, knowledge, and traditions.

Although the experience gained by the two analyzed HOs identifies a common evolutionary path in cultural heritage governance models—which doctrine had already observed (29)—in the opinion of the authors, the study conducted in this work deserves to be extended to other organizations with different characteristics. In particular, a potential direction for future research is considered interesting in governance model for hospital settings that possess specific museum or pictorial collections inherited from their past, yet disconnected from their current hospital context. Similarly, it would be worthwhile to conduct the study in settings where the ancient hospital has been abandoned for healthcare activities—which were transferred to a new facility—and is now entirely dedicated to cultural activities.

## Data Availability

The datasets generated and analyzed during the current study are available from the corresponding author upon request.

## Footnotes

1 According to UNESCO “Heritage conservation involves the protection and preservation of cultural heritage, including historic sites, artifacts, and traditions. This field emphasizes maintaining the integrity and significance of cultural resources for future generations. By promoting awareness and sustainable practices, heritage conservation ensures that diverse cultural narratives and identities continue to thrive in an ever-changing world”.

2 While cultural heritage's promotion is the whole of several and different action deputed to enhance the impact of cultural heritage's conservation within communities (20).

3 In Italy, pursuant to Article 1 of Decree-Law No. 83 of May 31, 2014, “Urgent measures for the protection of cultural heritage, the development of culture and the revitalization of tourism”, converted with amendments into Law No. 106 of July 29, 2014, as subsequently amended, a tax credit was introduced for monetary donations in support of culture and the performing arts, the so-called Art Bonus, as a form of patronage in favor of cultural heritage. Those who make monetary donations in support of culture, as provided by law, may benefit from significant tax advantages in the form of a tax credit.
